# Consumption of Yoghurt and Other Dairy Products and Risk of Colorectal Cancer in Iran: The IROPICAN Study

**DOI:** 10.3390/nu14122506

**Published:** 2022-06-16

**Authors:** Giulia Collatuzzo, Monireh Sadat Seyyedsalehi, Abbas Rezaeianzadeh, Maryam Marzban, Hamideh Rashidian, Maryam Hadji, Farin Kamangar, Arash Etemadi, Eero Pukkala, Kazem Zendehdel, Paolo Boffetta

**Affiliations:** 1 Department of Medical and Surgical Sciences, University of Bologna, 40138 Bologna, Italy; giulia.collatuzzo@studio.unibo.it (G.C.); monireh.seyydsalehi@unibo.it (M.S.S.); 2 Cancer Research Center, Cancer Institute, Tehran University of Medical Sciences, Tehran 1416634793, Iran; hmdhrashidian@yahoo.com (H.R.); maryam.hadji@tuni.fi (M.H.); kzendeh@sina.tums.ac (K.Z.); 3 Colorectal Research Center, Shiraz University of Medical Sciences, Shiraz 7146864685, Iran; rezaiana@gmail.com; 4 Department of Public Health, School of Public Health, Bushehr University of Medical Science, Bushehr 7514763448, Iran; marzabanh@gmail.com; 5 Clinical Research Development Center, The Persian Gulf Martyrs, Bushehr University of Medical Science, Bushehr 7514763448, Iran; 6 Health Sciences Unit, Faculty of Social Sciences, Tampere University, 33100 Tampere, Finland; eero.pukkala@cancer.fi; 7 Department of Biology, School of Computer, Mathematical, and Natural Sciences, Morgan State University, Baltimore, MD 21251, USA; farinkamangar@gmail.com; 8 Digestive Oncology Research Center, Digestive Diseases Research Institute, Shariati Hospital, Tehran University of Medical Sciences, Tehran 1416634793, Iran; arash.etemadi@nih.gov; 9 Metabolic Epidemiology Branch, Division of Cancer Epidemiology and Genetics, National Cancer Institute, Bethesda, MD 20810, USA; 10 Finnish Cancer Registry—Institute for Statistical and Epidemiological Cancer Research, 00100 Helsinki, Finland; 11 Stony Brook Cancer Center, Stony Brook University, Stony Brook, NY 11794, USA

**Keywords:** yoghurt, dairy products, colon, colorectal cancer, diet, nutrition epidemiology

## Abstract

Background: There is evidence of an inverse association between yoghurt intake and risk of colorectal cancer (CRC). We aimed at investigating the association between the intake of yoghurt and other dairy foods consumed in Iran and CRC risk. Methods: Our analysis included 4070 subjects within the IROPICAN (Iran Study of Opium and Cancer) study. Detailed information was collected by the use of validated questionnaires. We estimated adjusted odds ratios (OR) and 95% confidence intervals (CI) for the association between the intake of total dairy products, and, separately, of yoghurt, milk, cheese, kashk, dough, cream, ice cream, and other milk products, and CRC using unconditional logistic regression analyses. The intake was categorized in tertiles. Results: Overall, we analyzed 865 cases and 3205 controls. Total dairy products intake was not associated with CRC. The OR for one tertile increase (OR_T) in yoghurt intake was 0.97 (95% CI 0.87–1.08) for CRC and 0.66 (95% CI 0.52–0.84) for proximal colon cancer. Cream intake was associated with CRC (OR_T3 = 1.33, 95% CI 1.08–1.64), colon (OR_T3 = 1.37, 95% CI 1.03–1.81), and proximal cancer (OR_T3 = 1.29, 95% CI 1.04–1.61). The OR of distal colon cancer for ice cream intake was 0.59 (95% CI 0.43–0.82). Other dairy products were not associated with CRC risk.

## 1. Introduction

Worldwide, colorectal cancer (CRC) is the third leading incident cancer and the second leading cause of cancer death [1]. In Iran, CRC is the third most common cancer, with an estimated age-standardized incidence rate (ASR) of 15.9/100,000 for males and 11.9/100,00 for females [1,2]. Deepening the epidemiology of CRC is important given the burden of this neoplasm and its different patterns of distribution between high-income (29% in males, 20% in females) and low-middle-income countries (LMICs) (7.4% in males, 5.4% in females), the LMICs observing a worrying increase in CRC incidence [1].

These differences may be explained by several environmental and lifestyle habits. Diet is associated with CRC development, despite the magnitude of the association with the different food items being difficult to quantify [3]. The investigation of dietary factors is of major interest in medicine, given the possibility to modify them toward healthier patterns. The variety and complexity of foods may include factors that both increase or reduce the risk of cancer [4]. 

Several studies have investigated the role of dairy products in association with CRC. The recent analysis of evidence from a European cohort confirmed a decreased risk of CRC linked to dairy food and calcium [5]. Sun et al. recently reported an inverse relationship between yoghurt and CRC overall, despite the geographical stratification showing no significant association between dairy and CRC based on Asian studies [6].

Other authors found that the consumption of fermented dairy foods was inversely related to CRC precursors with a dose–response relationship [7,8]. However, the largest part of this research was conducted on European and American populations. Yoghurt in particular may exert an anti-cancer action on colon and rectum mucosa based on its probiotic components [9]. The composition and types of yoghurt can differ by country. Faghfoori and coauthors assessed the prophylactic effects of probiotic bacteria isolated from traditional dairy products consumed in Iran [10] by downregulating ErbB-2 and ErbB-3 gene expression. Indeed, dough, which is a typical dairy product consumed in Iran and contains yoghurt, water, salt, and herbs [11], has been proposed for its healthy properties, including against CRC, being rich in Lactobacillus fermentum [12].

We aimed at exploring the role of several dairy products, with emphasis on yoghurt, in an Iranian population with specific dietary and lifestyle habits, such as low red meat consumption and the use of opium. We aimed at describing the effect of different traditional Iranian dairy products, such as kashk [11] and dough, which have rarely been studied in association with CRC. 

## 2. Methods

### 2.1. Study Design and Population

This analysis is based on the IROPICAN study, a multicenter case–control study conducted in ten provinces of Iran, that was set up to investigate the association between opium use and risk of lung, colorectal, bladder, and head and neck cancers. Detailed information about the study protocol is provided elsewhere [13]. In the current analysis, we used incident primary colorectal cancer cases referred to the main cancer clinics and hospitals in seven provinces; most of the cases were pathologically confirmed from May 2017 to July 2020. Cases were defined using the International Classification of Diseases (ICD-O-3) codes as follows: proximal colon (C180–C185), distal colon (C186–C187), overlapping lesion of the colon (C188), not otherwise specified (C189), and rectum (C19–C20). Cases were frequency matched to cancer-free controls that were healthy hospital visitors to patients other than the cases at the date of recruitment by gender, age, and residential places of each case [14]. Participants who did not complete the food frequency questionnaire (FFQ) were excluded (*n* = 61). Anal cancer cases were also excluded from the analysis (*n* = 25). Finally, 865 cases and 3205 controls were included in the current study.

### 2.2. Dietary Assessment

The usual intake in the last year of 131 food items, including 113 foods, 17 dietary supplements, and water, was assessed using a qualitative FFQ, which was administered by trained interviewers [15]. The FFQ contained 5 sections: name of food, portion size (it depended on the types of food), frequency of use (daily, weekly, monthly, or yearly), quantity/amount of consumption, and considerations (detailed questions asked about the type of food of interest that were specified for each of foods). For each food item, the reported frequency of consumption was converted to frequency per day and was multiplied by the standard portion size (grams) using household measures to calculate grams per day. Then the daily energy and nutrient intake were calculated using the food composition database developed for the Iranian population by using USDA food composition [16], West Asia food composition [17], and Bahrain food composition [18].

### 2.3. Assessment of Other Variables

In the face-to-face interviews, lifestyle questionnaires were used to collect information about age, gender, cigarette smoking, opium use, socioeconomic status (SES), perceived workload and physical activity, and the use of nonsteroidal anti-inflammatory drugs (NSAIDs). In most centers, the body weight and standing height of controls and patients were measured by trained professionals at the entry time to the study. These data were used to calculate body mass index (BMI, weight/height squared, kg/m^2^). In particular, body weight 1 year before cancer diagnosis was asked to the cases, while body weight at the time of the interview was asked to the controls.

### 2.4. Statistical Analyses

Descriptive statistics were used to compare the distribution of the baseline characteristics and intake of dietary factors of cases and controls. Continuous variables were summarized using means and standard deviation (±SD) and categorical variables were summarized by counts and percentages. We estimated adjusted odds ratios (OR) and 95% confidence intervals (CI) for the association between intake of total dairy products (grams/day), yoghurt, milk, cheese, kashk, dough, cream, ice cream, and other milk products and CRC risk using logistic regression analyses. The analyses were conducted on all CRC patients and then on patients according to the sub-site of origin of cancer, including colon, proximal colon, distal colon, and rectum. Tertiles were calculated based on the distribution of dairy product intake in the full case–control study, and continuous analyses were also run across tertiles. The ORs were adjusted by gender, age, province, BMI, smoking, opium use (never user, regular user, non-regular user), aspirin intake, SES (low/medium/high), physical activity (light/moderate/heavy), red and processed meat intake, fat intake, and fiber intake. The main model was further adjusted by conjugated linoleic acid (CLA), excluding other fats, to investigate the potential confounding derived from its content.

Based on age, gender, SES, aspirin intake, opium use, fiber intake, and red and processed meat intake, a stratified analysis was conducted whose results were assessed using the LR test. We applied the Bonferroni correction to account for multiple comparisons.

All analyses were performed in Stata14 (Stata Statistical Software: Release 14. College Station, TX, USA: Stata Corp LLC).

### 2.5. Ethical Considerations

The study was approved by the Ethics Committee of the National Institute of Medical Research Development (NIMAD) (Code: IR.NIMAD.REC.1394.027). All participants signed written informed consent to participate in the study.

## 3. Results

The analyses included 4070 subjects, of whom 865 were CRC cases and 3205 were controls. The main characteristics of the study population are shown in Table 1.

While about 40% of the controls were of high SES, a corresponding proportion (38%) of cases were of low SES, with this difference mainly being due to the difference within rectum (40% low vs. 33% high) rather than colon cases (36% both low and high SES). Cases were less often cigarette smokers than controls, and opium consumption showed small differences between the groups of users (9–14% were regular users, and 4–5% non-regular users). No differences between cases and controls were found for physical activity and BMI. Red meat intake was higher in cases than in controls (24.3 vs. 18.6 g/day), with small difference according to cancer site (24.2 in colon vs. 24 in rectum cancer cases) (Table 2). There was a very mild difference in mean fiber intake between CRC cases (25.8 g/day) and controls (24.7 g/day). CRC cases reported a higher intake of total fat than controls (77.4 vs. 68.5 g/day), particularly colon compared to rectum cancer cases (79.4 vs. 74.2 g/day).

The total amount of dairy products consumed was higher among cases than controls, especially considering colon cancer cases (372.7 g/day vs. 337.4 g/day). When focusing on individual dairy products, each of those considered in the analysis was consumed in larger amount by CRC cases than controls. In particular, colon cancer cases reported a higher intake of yoghurt (148 g/day) than rectum cases and controls (142 g/day); a higher intake of milk (109.5 g/day) compared to rectum cases (91.7 g/day) and controls (95.7 g/day); a higher intake of cheese (26.5 g/day) compared to rectum cases (21.12 g/day) and controls (23.5 g/day); and a higher intake of dough (72.5 g/day) compared to controls (62.7 g/day).

Table 3 shows the results of the multivariate logistic regression models. Total dairy product intake was not associated with CRC overall, nor with proximal or distal colon or rectum cancer separately. Subjects with a high intake of yoghurt had an OR of 0.96 (95% CI 0.77–1.20) of developing CRC. The difference between the univariate and the multivariate analysis was explained by confounding by province and other factors. When we applied the Bonferroni correction for multiple comparisons, only the OR of proximal colon for yogurt intake remained statistically significant at α = 0.0012.

We found an inverse relationship between yoghurt intake and proximal colon cancer, equal to an OR of 0.63 (95% CI 0.39–1.01) for the second tertile and 0.43 (95% CI 0.27–0.70) for the top tertile, corresponding to an OR = 0.66 (95% CI 0.52–0.84) when considering one tertile of increased intake. No association was reported for the other CRC sites. The top tertile of dough intake resulted in a non-significant increase in the risk of CRC (OR = 1.26, 95% CI 0.98–1.61), while the top tertile of cream intake was significantly associated with CRC in overall (OR = 1.33, 95% CI 1.08–1.64), colon (OR = 1.37, 95% CI 1.03–1.81), as well as proximal colon cancer (OR = 1.68, 95% CI 1.08–2.61). We found no association between dough intake and risk of distal colon and rectal cancer. Increasing intake of ice cream was associated with a reduced risk of distal colon cancer (OR = 0.55, 95% CI 0.36–0.85 for second tertile; OR = 0.44, 95% CI 0.23–0.85 for top tertile of intake) but not for other sub-sites. Milk, cheese, kashk, and other milk products were not associated with any of the cancer sites.

When adjusting for CLA without other fats, the association between total dairy products (OR = 1.30, 1.02–1.65) and yoghurt (OR = 0.69, 0.55–0.87) and proximal colon cancer were largely overlapping with the corresponding ORs of the main analyses (not shown in detail).

The results of the stratified analysis by CRC determinants are shown in Table 4. Yoghurt appeared to be inversely related to CRC cancer, especially among subjects <50 years of age, non-aspirin users, subjects of low SES, and those with high fiber intake, although the interaction terms were not statistically significant. The association between yoghurt intake and risk of proximal colon cancer was also observed in the age category of <50 years (OR = 0.57, 95% CI 0.35–0.91). No other dairy variable was associated with any of the CRC anatomical sites in the younger age groups.

SES = socioeconomic status; ASA = acetylsalicylic acid; OR = Odds ratio; CI = confidence interval; SD= standard deviation.

## 4. Discussion

This study analyzes the effect of the intake of yoghurt and other dairy products on CRC risk in a population from different provinces of Iran. We showed an inverse association between yoghurt consumption and proximal colon cancer, with a dose–response relationship. High intake of cream was found to increase the risk of CRC, colon, and proximal colon cancer, while ice cream consumption appeared to be associated with a reduced risk of distal colon cancer. Other dairy products were not associated with CRC and its subsites.

While only the association between yogurt intake and risk of proximal colon remained statistically significant after application of Bonferroni correction, we think this approach is overly penalizing because the dairy factors and the outcomes included in the analysis were not mutually independent. An α level in the order of 0.005 is probably more appropriate to identify the associations which cannot confidently be attributed to chance.

We observed that yoghurt exerts an effect on proximal colon cancer, with a 0.44 OR among high vs. low consumers. An analysis of two cohort studies by Michels et al. obtained very similar results [19]: the authors reported an inverse relationship between yoghurt and proximal cancer with an OR of 0.84 (95% CI 0.71–1.00) for 1≥ servings/week compared to never/<1 serving a month among women and not men, with the standard serving corresponding to one 8 oz cup (237 mL). The mean consumption of yoghurt in our population was about 150 g/day, and the top tertile of consumption included subjects reporting >150 g/day, up to 460, which would correspond to a consumption comparable to that in Michel’s study [19]. As the test for trend was significant in Michel’s study and in ours, it can be assumed that a higher intake of yoghurt, more similar to that registered in our study population, would have been associated with an even lower risk of cancer of proximal colon in Michel’s cohorts [19]. A review from 2003 described the consistent protective effect of total dairy product intake and milk intake on CRC risk in cohort studies—and not in case–control ones—in the order of 40% and 20%, respectively [20]. A following meta-analysis published in 2009 confirmed these findings and calculated an RR of 0.84 (95% CI = 0.75–0.95) for total dairy products and of 0.78 (95% CI = 0.67–0.92) for milk intake on colon cancer. Traditional Moroccan dairy products have been investigated in association with CRC by El Kinany and coworkers in a five-center case–control study, evidencing how both common (milk and yoghurt) and traditional (lben, jben, raib—mainly from raw, boiled, and heated cow’s milk, fermented into curd) dairies inversely correlate with colon and rectal cancer and with a similar magnitude of effect (around 20% of reduced risk) [21].

The colon has functionally distinct regions, where the cecum and proximal regions of the colon are the major sites of fermentation, and the distal colon primarily extracts fluid and electrolytes (∼1.3 L/day). The proximal and distal colon are colonized by different functional subsystems of microbes, which are implied in the production of short-chain fatty acids, the conversion of primary bile acid to secondary bile acid, and the regulation of colon motility. The antioxidant activity in fermented dairy products is mainly due to the bioactive peptides released from α-lactalbumin, β-lactoglobulin, and α-casein [22].

A selective prophylactic action of yoghurt on the proximal colon may be connected to the growth of beneficial bacteria, which supports immune function and modulates inflammation, and to the long latency period for the development of the cancer from this anatomical site. Indeed, the association found by Michel and coauthors referred to a 16–20-year latency between yoghurt consumption and CRC incidence, underlying yoghurt’s potential in the primary prevention of this cancer. This is also consistent with the evidence of the gut microbiota acting in the early stages of colon carcinogenesis [23], emphasizing the potential benefit of fermented food against cancer development, especially in those sites of the colon where bacteria mostly exert their function [24]. Indeed, different compositions of gut microbiota have been described in relation to different CRC types and outcomes [25,26]. Our data refer to the year before CRC diagnosis/interview, used as a proxy for usual diet of the participants who could have changed their habits once aware of the disease or because of symptoms [27].

The role of yoghurt as source of health-promoting bacteria has been reviewed [28]. One way to redress or correct dysbiosis is via the ingestion of probiotics, fermented foods, and other dietary sources of beneficial microbes. The benefit conferred by a diet high in yoghurt consumption seemed independent from a generally healthier diet and lifestyle [28].

The composition of yoghurt is also important. Results from several randomized controlled trials have shown that probiotic yoghurts are generally more effective than conventional yoghurts for improving various health outcomes. A randomized control trial by Odamaki et al. evidenced the persistence of the baseline gut microbiota in subjects administered with 200 g of yoghurt supplemented with probiotics and meat-based diet for 5 days, while as many subjects eating simple yoghurt, as well as controls not receiving any type of yoghurt, and following the same meat-based diet underwent microbiota modification in a less healthy direction [29].

Barriers and facilitators to dairy product consumption in Iran has been reviewed by Rabiei et al., identifying a decreasing trend and a different pattern of consumption by age category [30]. When considering the improvement in dairy product composition from a health and nutritional viewpoint, cost should be taken into account. Indeed, cost is one of the barriers to the consumption of healthy foods, which are commonly more expensive [30].

Information on sweet additives (e.g., sugar or flavoring components) was not available. The addition of artificial sweeteners may indeed alter the effect of yoghurt and may represent an unhealthy factor [31,32]. Indeed, several studies have described the efforts made by food industries to reduce sugary content in yoghurt [33]. To date, no evidence is available with regard to the possible association between artificial sweeteners and CRC. If they were carcinogenic, given the lack of adjustment for this potential confounder, the inverse relation we found for yoghurt and proximal colon may be partially underestimated. In any case, Iranians mainly consume raw and more healthy yoghurt [34]—next to dough and kashk, which we considered as separate types of milk-based products. Sweet additives and CRC risk could be the object of future investigation. Detailed data on animal source of dairy products, as well as type of animal breeding and forage, were not available. The composition of milk can differ based on the species of origin, as well as nutritional and health properties [35]. This information may have helped in stratifying the risk between yoghurt and other dairy products and CRC.

The effect of yoghurt may be mediated by microbiota modification in a healthier direction. This is consistently described for yoghurt, with less evidence for other dairy products [36], partially explaining why results for other dairy products—mainly the non-fermented ones—are less consistent [27], thus resulting in the categorization of dairy products into specific food groups [37].

A balanced diet includes a number of important molecules from food, such as prebiotics, probiotics, antioxidants, polyunsaturated fatty acids, and isoflavones. The knowledge of the functions helpful for proper health balance is the basis of the concept of “functional food” [38].

The different results we found for each dairy food item and by anatomical cancer site stress the importance of addressing dairy food and CRC epidemiology through detailed stratification.

The analysis of separate dairy products allowed us to underline the different roles of each of them in terms of the direction and magnitude of the association. In particular, the association with CRC was positive for dough, kashk, and cream.

Dough and kashk are traditional fermented dairy foods in Iran. Potential contaminants, including aflatoxins, have been observed in white and cream cheese in Iran [39], in addition to evidence of seasonal variation in this risk [40,41], which remained lower in yoghurt than other dairy products [42]. A recent review has shown that 89% of dairy products exceeded the standard limits of aflatoxins in Iran, in particular 17.8% of cheese, 14% of yoghurt, 12.63% of kashk, and 2.1% of dough. These results would suggest lower burden attributable to kashk and dough rather than yoghurt [42].

The amount of saturated fat in dairy products is another contributing factor in relation to CRC cancer [20]. An ecological study in Iran showed that high-fat dairy product intake may have a positive association with CRC risk [43]. Among the possible mechanisms under the effect of dairy products on CRC, those based on fatty acids content have been widely described [44,45]. Dairy foods are particularly rich in CLA, whose properties include anti-cancer effects through apoptosis, as well as the inhibition of inflammation and neo-angiogenesis [46]. Neither total fats nor CLA confound the inverse association identified between total dairies and yoghurt and proximal colon cancer, suggesting an independent role of the exposures in the effect exerted.

Cream is a common part of Iranian breakfast. To our best knowledge, no study has measured the association between cream and CRC in Iran, and the literature reports increased overall cancer mortality with high-fat dairy product intake and low cancer mortality with low-fat dairy intake [47], but no relation with CRC in particular [47]. All in all, the results on fatty dairies remain inconsistent, as they are shown to take opposite directions in different studies [48,49]. Our findings may sustain the recommendation of limiting high-fat dairy product consumption, which belongs to an unhealthy dietary pattern [50]. Cheese is also commonly eaten for breakfast in Iran and contains a high amount of salt (Lighvan cheese, Feta cheese). In addition, salt is added as part of the preparation of dough and kashk. They are dairy products with high salt content, compared to yoghurt and other milk products. There has been evidence that salt-containing foods can be related to CRC in several previous studies [51,52].

The direction of our results—which were, in any case, not significant—does not agree with common findings on the healthy properties of dough. Dough is rich in Lactobacillus fermentum, whose [12] probiotic effects have been described and explored by strain, individuating those mostly promising in the perspective of possible application as novel probiotic starter cultures [53]. In addition, 3D cultures have shown the anti-cancer properties of Lactobacillus fermentum [54]. Conversely, a study conducted among 3846 Iranians, including 824 affected by irritable bowel syndrome, reported a tendency of avoiding the intake of dough due to the occurrence of GI symptoms compared to controls [55]. Despite this not being directly in support of a role in disease induction, the study may imply a negative effect of dough on the gut microbiota with the possibility of symptoms outburst and may also suggest an interrelation between dough consumption, microbiota, and mucosal health [56].

More than 200 participants in the IROPICAN cohort were diagnosed with CRC before 50 years of age, representing early onset cases. When restricting the analysis among subjects of this age group, neither yoghurt nor the other dairy products or dairy products overall were associated with CRC, overall colon, distal colon, and rectal cancer. Conversely, yoghurt resulted in being inversely related to proximal colon cancer, with an almost halved risk by tertile increase (*p* = 0.001); this relation remained when adjusting for all the other dairy products taken together, which were instead not related to the outcome. This result is valuable given the increasing trend of CRC diagnosed at a young age and the outmost importance of identifying possible factors associated with early cancer occurrence [57,58].

Similarly, we found a stronger inverse relationship between yoghurt and proximal cancer in males, subjects of low SES, and no aspirin users; no prevailing effect of yoghurt was found for low vs. high intake of fiber, while individuals with high red and processed meat consumption seemed to have major benefit from yoghurt.

Given the consistent evidence of reduced risk of proximal colon cancer in subjects used to a high consumption of yoghurt, and based on the heterogeneity in the results for other dairy products, attention should be paid to the composition of dairy products when addressing this topic in epidemiological studies. Following these considerations, and considering our results, low intake of dairy items with high salt and fat content may be recommended, in favor of fermented dairies and yoghurt in particular. In addition, new food technologies could take advantage of this evidence, focusing on the development of probiotic foods with a healthy nutritional components rate [59].

The present study is part of a larger case–control investigation of four different cancer types (lung, bladder, head and neck cancer, in addition to CRC), which could suffer from some biases such as selection bias and reporting bias, especially with regard to the FFQ because of memory dependence. Due to the nature of the main project, the controls were not chosen on a population-based approach but were taken among the healthy visitors who did not have CRC. This approach may be subject to selection bias. However, our validation study showed that due to appropriate matching and using healthy visitors instead of disease controls, such bias is minimal [14]. It was not possible to separate dairy products by fat content (low-fat, regular) or traditional and industrial types. In addition, we could not distinguish the animal source of the different dairies, nor the breeding conditions of the animals. Lastly, information on sugar or other additives was not available.

Among the study’s strengths are its large sample size, detailed data collection about dietary intake and potential confounders by interviewing trained investigators and supervisors, similar food albums and questioning tools across all centers, high participation rates among cases and controls, and histologic confirmation of cancer diagnoses by the pathological report. Information on diet was collected from cases at the moment of cancer diagnosis, with small likelihood of diet change. To our knowledge this is the first large study to explore the association between traditional Iranian dairy foods and CRC on a large geographical basis, and it is probably the largest in the Eastern Mediterranean region. Indeed, the multicentric design of the study allowed us to investigate a large number of dietary factors prevalent in different regions of the country.

## 5. Conclusions

In conclusion, we found an inverse relationship between yoghurt consumption and proximal colon cancer, with a dose–response effect. Dough and cream appeared to increase the risk of CRC, while ice cream was inversely related to distal colon cancer; we suggest interpreting these findings with caution, suggesting the role of dairy food components rather than a causal relationship between these specific foods. No association was found between total dairy products and CRC. The null result for dairy products overall seems to derive from the opposite direction of the effect exerted by different dairies, with dough and cream showing a positive association with CRC. Our results provide new data on the role of dairy foods consumed in Iran on CRC epidemiology and may add useful information for the development of functional dairy food.

Dairy food composition, e.g., high fat or salt content, may play a role in the association between dairy products and CRC.

## Figures and Tables

**Table 1:** Selected baseline demographic and lifestyle characteristics of study participants by colorectal cancer status, IROPICAN study.

	Controls	Cases		
		Colorectal *	Colon	Rectum
**Overall**	3205	865	434	404
**Province, N (%)**				
Tehran	816 (25.46)	165 (19.08)	101 (23.27)	64 (15.84)
Fars	943 (29.42)	248 (28.67)	93 (21.43)	155 (38.37)
Kerman	525 (16.38)	100 (11.56)	49 (11.29)	51 (12.62)
Golestan	372 (11.61)	149 (17.23)	89 (20.51)	53 (13.12)
Mazandaran	136 (4.24)	59 (6.82)	34 (7.83)	25 (6.19)
Kermanshah	251 (7.83)	68 (7.86)	31 (7.14)	35 (8.66)
Mashhad	162 (5.05)	76 (8.79)	37 (8.53)	21 (5.20)
**Gender, N (%)**				
Women	1002 (31.26)	368 (42.54)	193 (44.47)	169 (41.83)
Men	2203 (68.74)	497 (57.46)	241 (55.53)	235 (58.17)
**Age, years, mean (±SD)**	57.18 (11.49)	58.62 (12.44)	58.78 (12.29)	58.3 (12.61)
**Age, years, N (%)**				
<50	751 (23.43)	193 (22.31)	99 (22.81)	89 (22.03)
≥50 and <60	993 (30.98)	242 (27.98)	112 (25.81)	123 (30.45)
≥60 and <70	1019 (31.79)	258 (29.83)	137 (31.57)	112 (27.72)
≥70	442 (13.79)	172 (19.88)	86 (19.82)	80 (19.80)
**Socio-economic status (SES), N (%)**				
Low	860 (26.83)	331 (38.27)	159 (36.64)	161 (39.85)
Moderate	1078 (33.63)	234 (27.05)	118 (27.19)	109 (26.98)
High	1267 (39.53)	300 (34.68)	157 (36.18)	134 (33.17)
**Tobacco consumption, N (%)**				
No	2280 (71.14)	677 (78.27)	356 (82.03)	300 (74.26)
Yes	925 (28.86)	188 (21.73)	78 (17.97)	104 (25.74)
**Opium consumption, N (%)**				
Never use	2639 (82.34)	738 (85.32)	373 (85.94)	343 (84.90)
Regular user	439 (13.70)	89 (10.29)	40 (9.22)	47 (11.63)
Non-regular user	127 (3.96)	38 (4.39)	21 (4.84)	14 (3.47)
**Workload Physical activity, N (%)**				
Sedentary	1033 (32.27%)	287 (33.18%)	147 (33.87%)	132 (32.67%)
Moderate	701 (21.88%)	155 (17.92%)	78 (17.97%)	72 (17.82%)
Heavy	694 (21.66%)	184 (21.27%)	87 (20.05%)	87 (21.53%)
Unknown	775 (24.19%)	239 (27.63%)	122 (28.11%)	113 (27.97%)
**BMI, kg/m^2^, mean (±SD)**	26.59 (4.72)	26.93 (4.99)	26.91 (5.07)	26.83 (4.85)

* 27 cases with unknown site within the colorectum, BMI; body mass index.

**Table 2:** Mean Dietary factors at baseline among control and colorectal case subjects in the IROPICAN Study.

Dietary Intake, g/day, Mean (SD)	Controls N = 3205	Cases		
		Colorectal * N = 865	Colon N = 434	Rectum N = 404
**Red and processed meat**	18.66 (19.29)	24.30 (27.06)	24.26 (26.48)	24.00 (27.65)
Fiber	24.72 (11.25)	25.86 (12.38)	25.28 (12.16)	26.34 (12.73)
Total fat	68.53 (29.92)	77.39 (39.70)	79.40 (39.71)	74.19 (39.29)
**Dietary intakes of Dairy Products, g/day, mean (SD)**				
Total dairy products	337.40 (193.26)	362.49 (214.98)	372.72 (217.27)	341.14 (203.38)
Yoghurt	142.98 (93.71)	148.23 (98.86)	148.89 (99.44)	142.18 (94.58)
Milk	95.72 (96.73)	101.69 (103.66)	109.45 (103.58)	91.74 (102.74)
Cheeses	23.48 (19.99)	24.03 (21.04)	26.50 (23.63)	21.15 (17.76)
Kashk	3.20 (9.07)	4.92 (12.43)	5.26 (13.76)	4.48 (10.96)
Dough	62.65 (75.45)	72.54 (83.93)	70.52 (80.96)	72.22 (82.70)
Cream	1.01 (3.25)	1.52 (4.08)	1.56 (4.47)	1.31 (3.35)
Ice cream	4.66 (7.70)	5.03 (9.75)	4.92 (7.89)	5.16 (11.33)
Other milk products *	4.25 (24.25)	6.41 (28.14)	6.95 (30.39)	5.37 (23.60)

* Other milk: including different industrial milk products (banana milk, chocolate milk, and other).

**Table 3:** Dietary estimates of dairy products and risk of colorectal cancer and subtypes (colorectal/colon/proximal colon/distal colon/rectum). Reference category: tertile 1.

Dairy Products Types	Cancer Type	Mean (g/Day)	T2 OR (95%CI)	Mean (g/Day)	T3 OR (95% CI)	OR (95% CI) (For One Tertile Increase)	*p*-Value (For One Tertile Increase)	*p*-Value (Continuous)
**Total Dairy products**	**Colorectal Cancer**	313.17	1.00 (0.81–1.22)	562.87	1.06 (0.85–1.32)	1.03 (0.92–1.15)	0.557	0.293
	**Colon Cancer**	320.87	0.86 (0.65–1.13)	577.42	1.00 (0.75–1.34)	1.00 (0.87–1.16)	0.911	0.661
	**Proximal colon**	317.463	0.82 (0.51–1.31)	559.76	0.98 (0.61–1.58)	1.00 (0.79–1.28)	0.974	0.820
	**Distal colon**	322.59	0.98 (0.66–1.46)	569.40	0.96 (0.62–1.47)	0.98 (0.79–1.20)	0.858	0.941
	**Rectum Cancer**	314.28	1.11 (0.85–1.45)	584.86	1.06 (0.78–1.44)	1.03 (0.88–1.20)	0.671	0.461
***Yoghurt***	***Colorectal Cancer***	110.30	1.06 (0.86–1.32)	238.69	0.96 (0.77–1.20)	0.97 (0.87–1.08)	0.629	0.450
	***Colon Cancer***	109.37	1.12 (0.83–1.49)	244.72	0.78 (0.58–1.06)	0.87 (0.76–1.01)	0.074	*0.042*
	***Proximal colon***	108.31	0.63 (0.39–1.01)	250.56	*0.43 (0.27–0.70)*	*0.66 (0.52–0.84)*	*0.001*	*0.007*
	***Distal colon***	110.66	1.27 (0.83–1.94)	238.47	0.81 (0.52–1.26)	0.88 (0.71–1.08)	0.259	0.085
	***Rectum Cancer***	109.14	0.97 (0.72–1.30)	237.89	1.07 (0.80–1.45)	1.03 (0.88–1.19)	0.671	0.819
**Milk**	**Colorectal Cancer**	90.30	0.84 (0.69–1.01)	242.22	0.98 (0.79–1.21)	0.97 (0.88–1.09)	0.708	0.848
	**Colon Cancer**	93.13	0.95 (0.73–1.23)	244.47	1.06 (0.80–1.41)	1.03 (0.90–1.20)	0.596	0.632
	**Proximal colon**	96.48	1.11 (0.72–1.70)	245.28	1.18 (0.74–1.88)	1.10 (0.88–1.40)	0.406	0.394
	**Distal colon**	90.16	0.97 (0.66–1.42)	232.16	1.30 (0.87–1.96)	1.14 (0.93–1.39)	0.202	0.374
	**Rectum Cancer**	86.71	0.75 (0.58–0.98)	247.17	0.97 (0.72–1.31)	0.95 (0.82–1.10)	0.531	0.741
***Dough***	***Colorectal Cancer***	59.18	1.17 (0.97–1.42)	197.08	1.26 (0.98–1.61)	*1.14 (1.01–1.29)*	*0.025*	*0.034*
	***Colon Cancer***	63.55	1.04 (0.81–1.33)	198.23	1.15 (0.83–1.60)	1.09 (0.93–1.28)	0.289	0.165
	***Proximal colon***	63.03	1.21 (0.81–1.83)	174.45	1.52 (0.88–2.61)	1.23 (0.94–1.61)	0.120	0.368
	***Distal colon***	61.64	1.02 (0.71–1.46)	197.91	1.06 (0.65–1.73)	1.05 (0.83–1.33)	0.684	0.554
	***Rectum Cancer***	61.06	1.28 (0.99–1.65)	200.07	1.36 (0.96–1.91)	*1.18 (1.00–1.40)*	*0.041*	0.092
***Kashk***	***Colorectal Cancer***	1.165	*0.70 (0.57–0.85)*	11.58	1.03 (0.81–1.31)	0.99 (0.88–1.13)	0.949	*0.005*
	**Colon Cancer**	1.13	0.66 (0.50–0.87)	14.08	1.09 (0.79–1.49)	1.01 (0.86–1.20)	0.856	*0.019*
	**Proximal colon**	1.16	0.79 (0.51–1.21)	10.38	0.90 (0.52–1.58)	0.93 (0.70–1.23)	0.625	0.514
	**Distal colon**	1.13	0.64 (0.43–0.94)	12.98	0.91 (0.57–1.44)	0.91 (0.72–1.17)	0.486	0.101
	**Rectum Cancer**	1.19	0.73 (0.55–0.97)	14.44	1.01 (0.73–1.40)	0.99 (0.84–1.18)	0.926	*0.068*
***Cheese***	***Colorectal Cancer***	26.73	0.89 (0.74–1.08)	63.27	1.08 (0.81–1.44)	0.98 (0.86–1.12)	0.849	0.946
	***Colon Cancer***	26.87	1.00 (0.78–1.29)	70.21	1.08 (0.74–1.56)	1.03 (0.87–1.24)	0.724	0.418
	***Proximal colon***	26.35	1.05 (0.70–1.57)	74.13	0.78 (0.40–1.49)	0.93 (0.70–1.25)	0.630	0.870
	***Distal colon***	26.99	0.94 (0.65–1.35)	69.57	1.20 (0.70–2.05)	1.05 (0.81–1.35)	0.675	0.448
	***Rectum Cancer***	25.79	*0.77 (0.60–0.99)*	57.30	0.96 (0.63–1.47)	0.90 (0.74–1.08)	0.299	0.119
***Cream***	***Colorectal Cancer***	*0.451*	1.07 (0.87–1.32)	4.61	*1.33 (1.08–1.64)*	*1.11 (1.01–1.24)*	*0.040*	0.317
	**Colon Cancer**	0.462	1.23 (0.94–1.61)	5.17	*1.37 (1.03–1.81)*	1.14 (1.00–1.31)	0.057	0.499
	**Proximal colon**	0.442	1.30 (0.83–2.04)	6.23	*1.68 (1.08–2.61)*	*1.29 (1.04–1.61)*	*0.021*	*0.040*
	**Distal colon**	0.41	1.07 (0.72–1.58)	4.97	0.93 (0.60–1.43)	0.93 (0.76–1.15)	0.527	0.516
	**Rectum Cancer**	0.465	0.99 (0.75–1.32)	4.99	1.20 (0.90–1.60)	1.05 (0.91–1.01)	0.478	0.741
***Ice cream***	***Colorectal Cancer***	3.07	0.83 (0.65–1.06)	22.28	0.86 (0.62–1.21)	0.88 (0.74–1.04)	0.160	0.777
	***Colon Cancer***	3.17	*0.72 (0.52–0.98)*	22.00	0.75 (0.48–1.17)	0.82 (0.64–1.02)	0.088	0.651
	***Proximal colon***	3.26	1.25 (0.67–2.34)	22.71	1.48 (0.68–3.22)	1.20 (0.82–1.77)	0.330	0.531
	***Distal colon***	*3.31*	*0.55 (0.36–0.85)*	*19.44*	*0.44 (0.23–0.85)*	*0.59 (0.43–0.82)*	*0.002*	0.108
	***Rectum Cancer***	3.16	0.92 (0.67–1.28)	22.02	0.98 (0.61–1.55)	0.95 (0.75–1.20)	0.719	0.400
**Other milk products**	**Colorectal Cancer**	*4.17*	*0.70 (0.53–0.93)*	*59.17*	1.00 (0.73–1.37)	0.91 (0.78–1.05)	0.204	0.233
	**Colon Cancer**	*4.18*	*0.66 (0.45–0.97)*	*83.54*	0.99 (0.65–1.50)	0.89 (0.64–1.02)	0.260	0.145
	**Proximal colon**	4.41	1.07 (0.61–1.87)	80.55	1.04 (0.53–2.03)	1.02 (0.75–1.38)	0.853	0.208
	**Distal colon**	*4.20*	*0.52 (0.28–0.95)*	*65.84*	1.07 (0.60–1.92)	0.90 (0.67–1.20)	0.493	0.641
	**Rectum Cancer**	5.04	0.77 (0.53–1.13)	62.91	0.96 (0.63–1.47)	0.91 (074–1.10)	0.372	0.685

Adjusted by gender, age, BMI, smoking, opium, province, aspirin, SES, physical activity, use of red and processed meat, fat intake, fiber intake.

**Table 4:** Odds ratio and 95% confidence interval of dairy products and colorectal cancer stratified by socioeconomic status (SES), aspirin use (ASA), age, sex, opium consumption, fiber intake, and red and processed meat intake.

Stratification Factors		Total Dairy Products without Yoghurt					Yoghurt				
		Colorectal or (95% CI)	Colon or (95% CI)	Proximal Colon or (95% CI)	Distal Colon or (95% CI)	Rectum or (95% CI)	Colorectal or (95% CI)	Colon or (95% CI)	Proximal Colon or (95% CI)	Distal Colon or (95% CI)	Rectum or (95% CI)
**SES**	**Low**	**1.23** **(1.02–1.48)**	1.26 (0.98–1.63)	1.45 (0.91–2.30)	1.32 (0.91–1.93)	1.25 (0.96–1.61)	0.90 (0.75–1.08)	0.80 (0.62–1.03)	0.57 (0.36–0.88)	0.83 (0.57–1.19)	0.95 (0.74–1.21)
	**Moderate**	0.99 (0.80–1.22)	1.08 (0.82–1.42)	1.02 (0.66–1.60)	1.20 (0.81–1.78)	0.95 (0.71–1.27)	0.90 (0.73–1.10)	0.75 (0.57–0.99)	0.75 (0.49–1.17)	0.67 (0.45–0.97)	1.02 (0.76–1.36)
	**High**	0.95 (0.79–1.15)	0.93 (0.73–1.19)	1.31 (0.89–1.93)	0.77 (0.54–1.10)	1.02 (0.79–1.33)	1.13 (0.94–1.36)	1.04 (0.32–1.33)	0.65 (0.45–0.95)	1.22 (0.85–1.77)	1.22 (0.94–1.57)
**ASA**	**No**	1.06 (0.94–1.20)	1.09 (0.93–1.28)	1.25 (0.95–1.63)	1.07 (0.84–1.35)	1.06 (0.90–1.26)	0.98 (0.87–1.11)	0.87 (0.74–1.01)	0.67 (0.52–0.87)	0.85 (0.67–1.07)	1.06 (0.90–1.24)
	**Yes**	1.02 (0.78–1.33)	1.10 (0.76–1.59)	1.59 (0.82–3.08)	1.00 (0.60–1.68)	0.99 (0.69–1.42)	0.93 (0.72–1.21)	0.89 (0.62–1.28)	0.61 (0.33–1.12)	1.06 (0.63–1.79)	0.93 (0.66–1.33)
**Age**	**≤50**	1.11 (0.89–1.39)	1.22 (0.90–1.64)	1.11 (0.67–1.82)	1.48 (0.94–2.33)	1.03 (0.76–1.41)	0.91 (0.74–1.13)	0.81 (0.61–1.08)	0.57 (0.35–0.91)	0.83 (0.53–1.28)	0.96 (0.71–1.30)
	**>50**	1.01 (0.90–1.16)	1.02 (0.86–1.21)	1.28 (0.96–1.69)	0.91 (0.71–1.16)	1.04 (0.88–1.24)	0.99 (0.88–1.13)	0.89 (0.75–1.05)	0.69 (0.53–0.91)	0.91 (0.72–1.16)	1.05 (0.88–1.25)
**Opium**	**No**	1.03 (0.91–1.16)	1.09 (0.93–1.28)	1.29 (0.99–1.69)	1.10 (0.87–1.36)	1.02 (0.86–1.20)	1.03 (0.91–1.15)	0.94 (0.80–1.09)	0.68 (0.53–0.88)	0.96 (0.76–1.19)	1.07 (0.90–1.24)
	**Yes**	1.16 (0.85–1.58)	0.97 (0.65–1.47)	1.13 (0.57–2.17)	1.04 (0.55–1.97)	1.32 (0.84–2.00)	0.77 (0.57–1.03)	0.59 (0.40–0.89)	0.43 (0.22–0.82)	0.52 (0.27–1.01)	0.86 (0.57–1.35)
**Fiber** **intake** **Mean (±SD)**	**13.17 (±3.02)**	1.07 (0.83–1.33)	1.18 (0.85–1.54)	1.24 (0.71–2.20)	1.15 (0.71–1.71)	0.97 (0.66–1.32)	0.99 (0.78–1.23)	0.97 (0.72–1.28)	0.54 (0.31–0.91)	1.03 (0.66–1.58)	1.00 (0.72–1.39)
	**20.08 (±1.60)**	1.14 (0.90–1.44)	1.29 (0.87–1.64)	1.28 (0.78–2.23)	1.17 (0.72–1.83)	1.14 (0.83–1.60)	1.01 (0.79–1.28)	0.83 (0.60–1.15)	0.63 (0.38–1.08)	0.90 (0.55–1.46)	1.13 (0.80–1.58)
	**26.18 (±2.18)**	1.06 (0.83–1.35)	1.06 (0.76–1.45)	1.29 (0.81–2.07)	0.90 (0.52–1.48)	1.11 (0.80–1.55)	1.18 (0.93–1.50)	0.89 (0.66–1.22)	0.75 (0.48–1.22)	0.85 (0.53–1.40)	1.47 (1.04–2.05)
	**40.39 (±10.21)**	1.12 (0.89–1.38)	1.18 (0.87–1.62)	1.46 (0.85–2.62)	1.38 (0.84–2.04)	1.11 (0.82–1.45)	0.79 (0.64–0.98)	0.72 (0.55–0.99)	0.50 (0.31–0.89)	0.66 (0.47–1.06)	0.82 (0.62–1.06)
**Red and processed meat** **Mean (±SD)**	**4.47 (±2.90)**	1.12 (0.93–1.34)	1.09 (0.80–1.43)	1.33 (0.81–2.24)	0.97 (0.81–1.54)	1.16 (0.72–1.30)	1.00 (0.84–1.19)	0.98 (0.63–1.10)	0.76 (0.30–1.80)	1.15 (0.77–1.45)	0.96 (0.83–1.52)
	**15.09 (±3.42)**	1.03 (0.82–1.29)	1.18 (0.85–1.64)	1.23 (0.80–2.21)	1.70 (1.03–2.79)	0.97 (0.72–1.30)	1.02 (0.81–1.2)	0.90 (0.63–1.10)	0.68 (0.56–1.44)	0.90 (0.52–1.21)	1.09 (0.84–1.52)
	**40.34 (±24.27)**	1.02 (0.84–1.23)	1.03 (0.80–1.32)	1.27 (0.83–1.94)	0.90 (0.63–1.27)	1.03 (0.79–1.33)	0.94 (0.78–1.12)	0.79 (0.63–1.01)	0.60 (0.40–0.88)	0.72 (0.52–1.01)	1.09 (0.85–1.41)

## Data Availability

The data presented in this study are available on request from the corresponding author. The data are not publicly available due to privacy reasons.
